# Supplementary figures and images for: Pinocembrin Downregulates Vascular Smooth Muscle Cells Proliferation and Migration Leading to Attenuate Neointima Formation in Balloon-Injured Rats
Source: Biomolecules. 2025 Sep 17;15(9):1325. doi: 10.3390/biom15091325 (PMC12467540; doi:10.3390/biom15091325)

# Original Images for Blots

Figure 1.

D

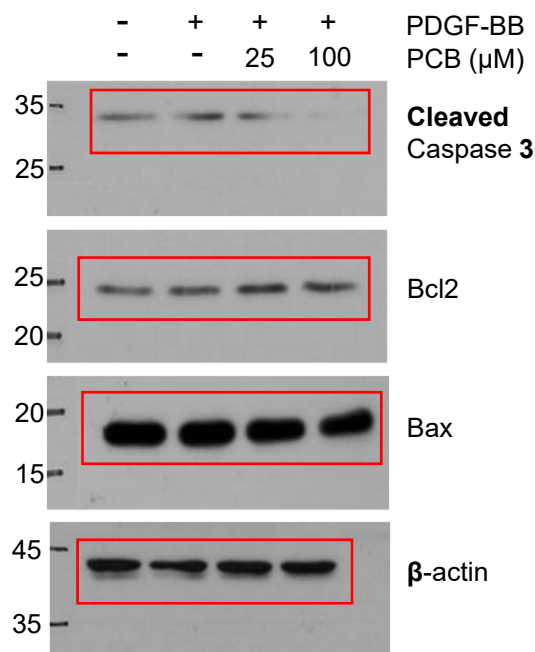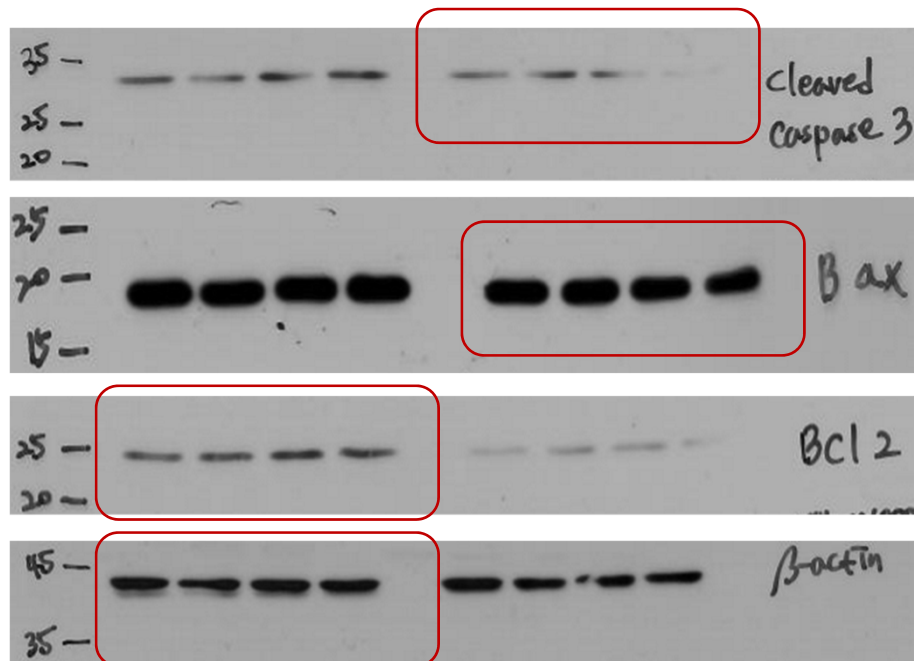

Figure 2.

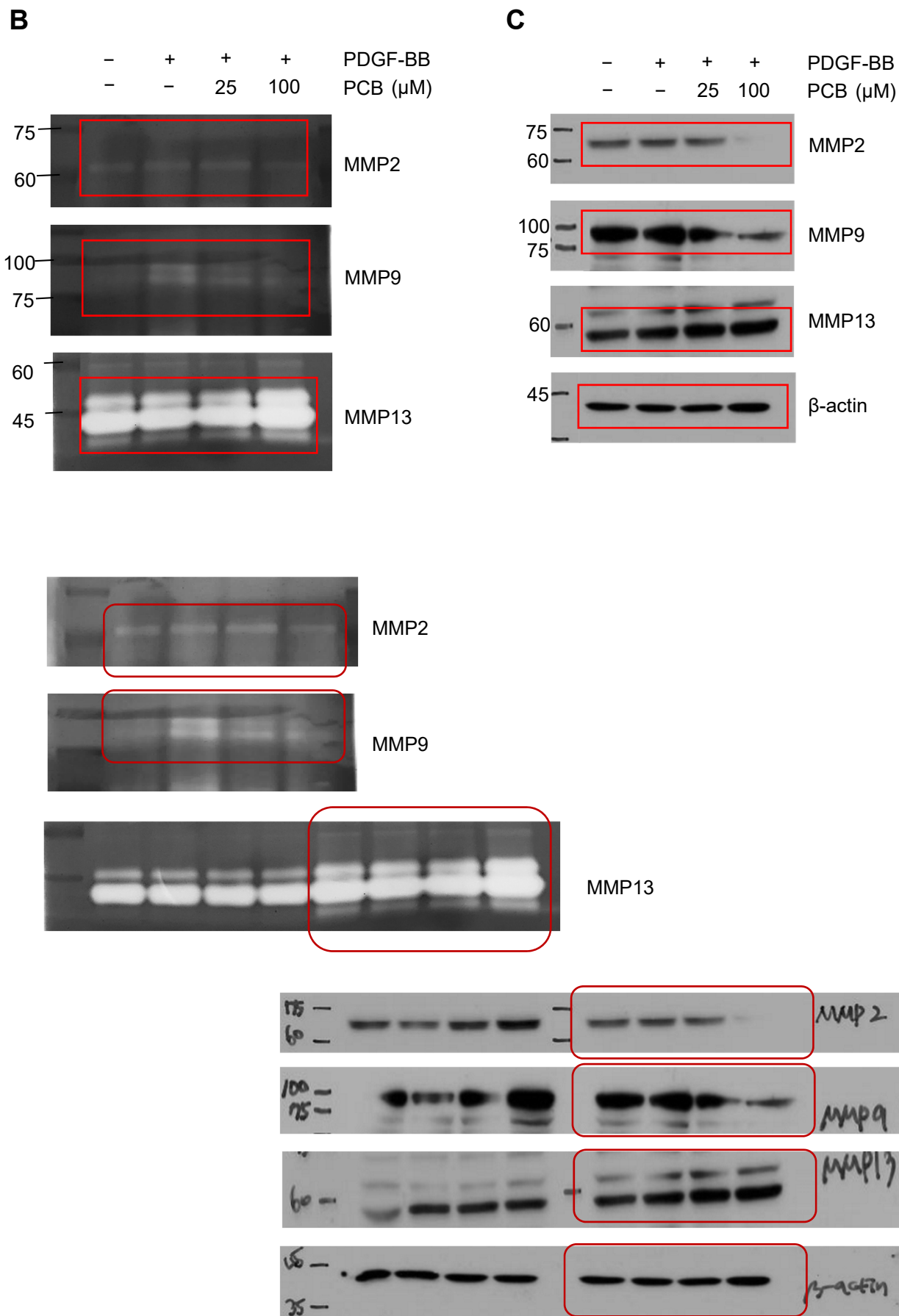

Figure 3.

C

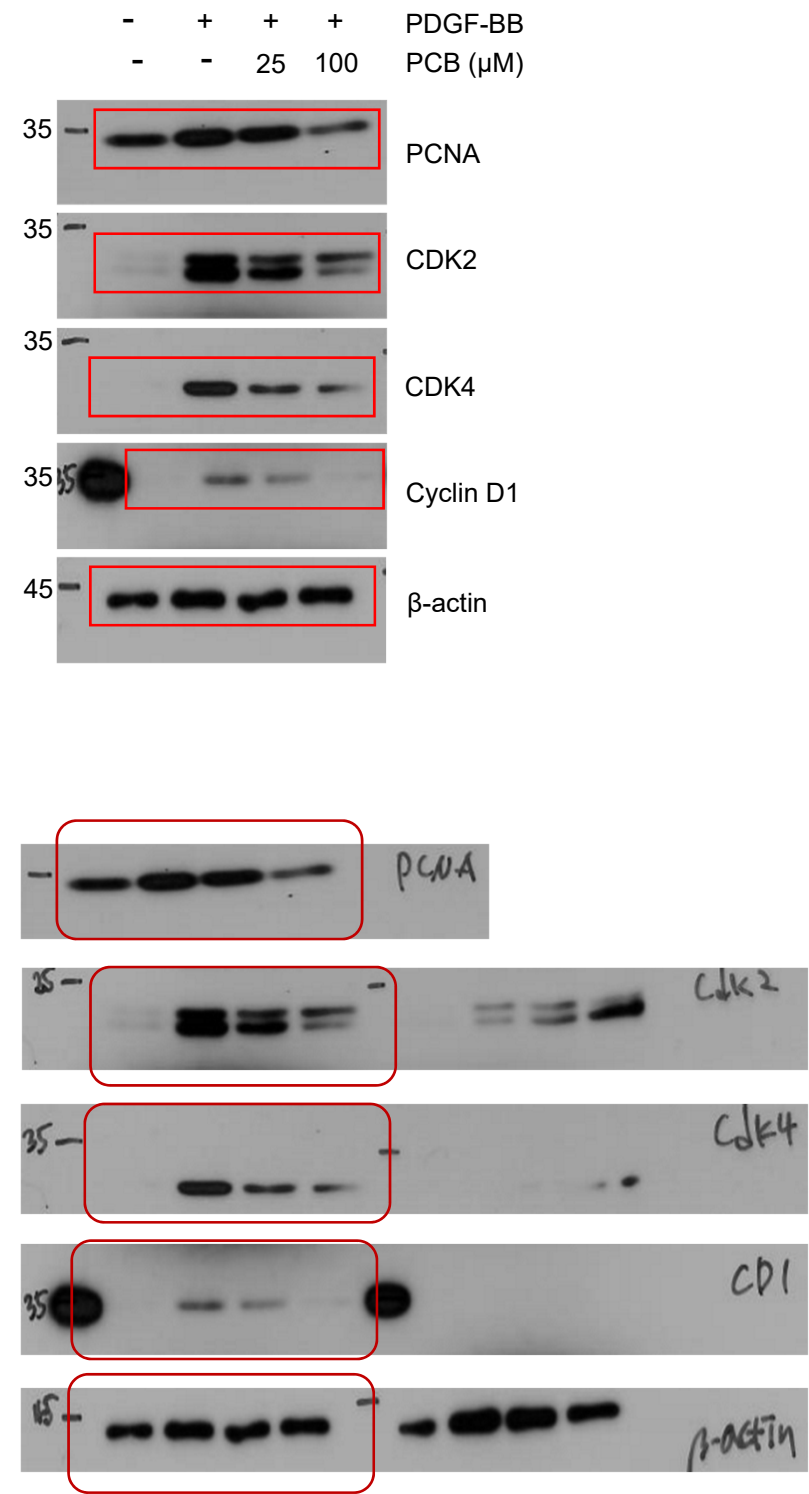

Figure 4.

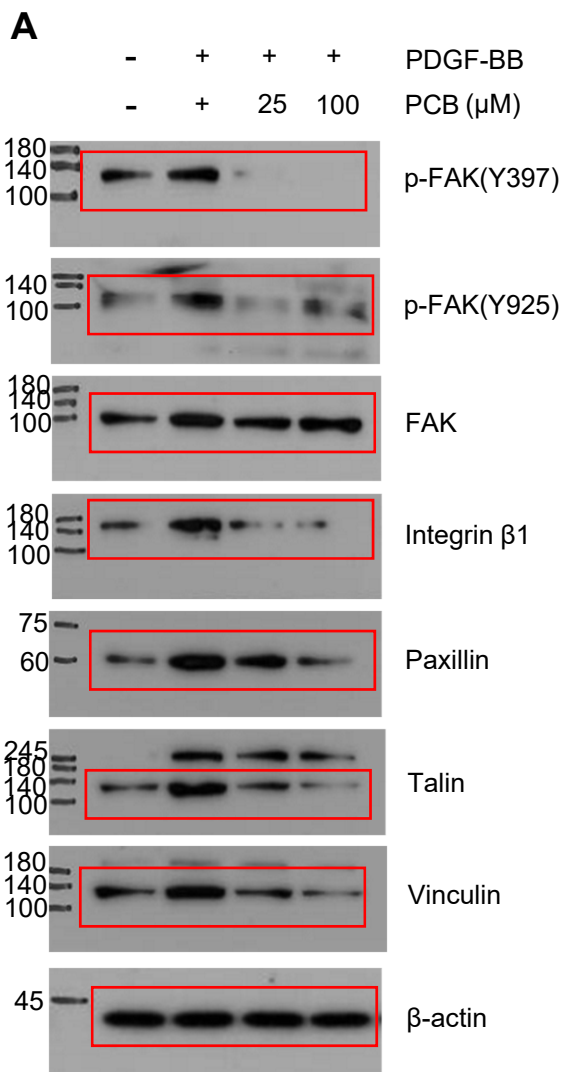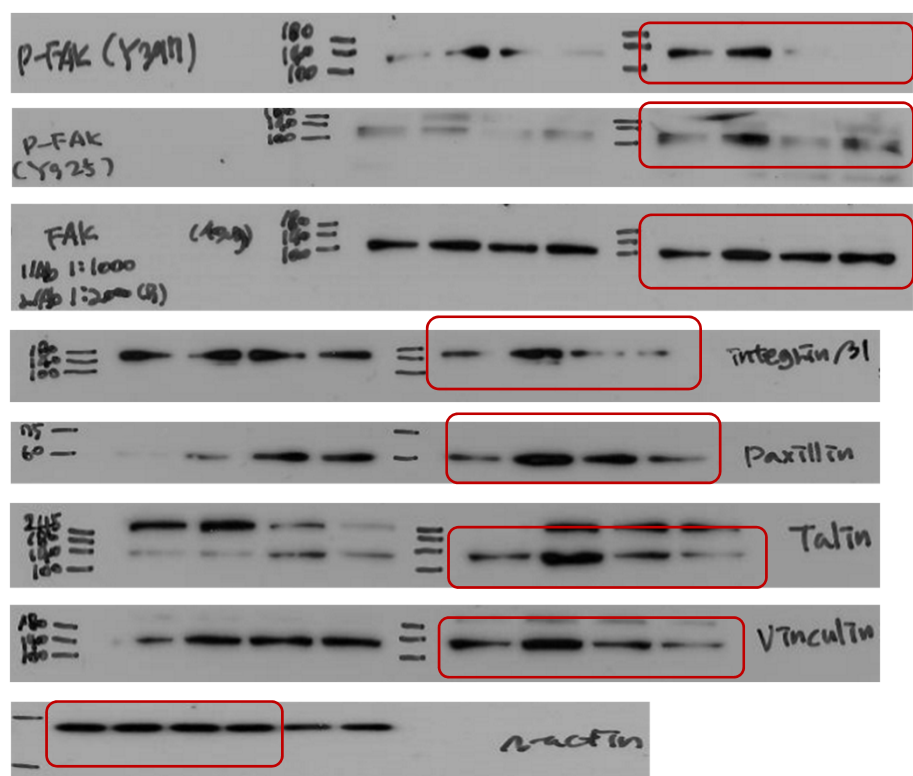

Figure 4.

C

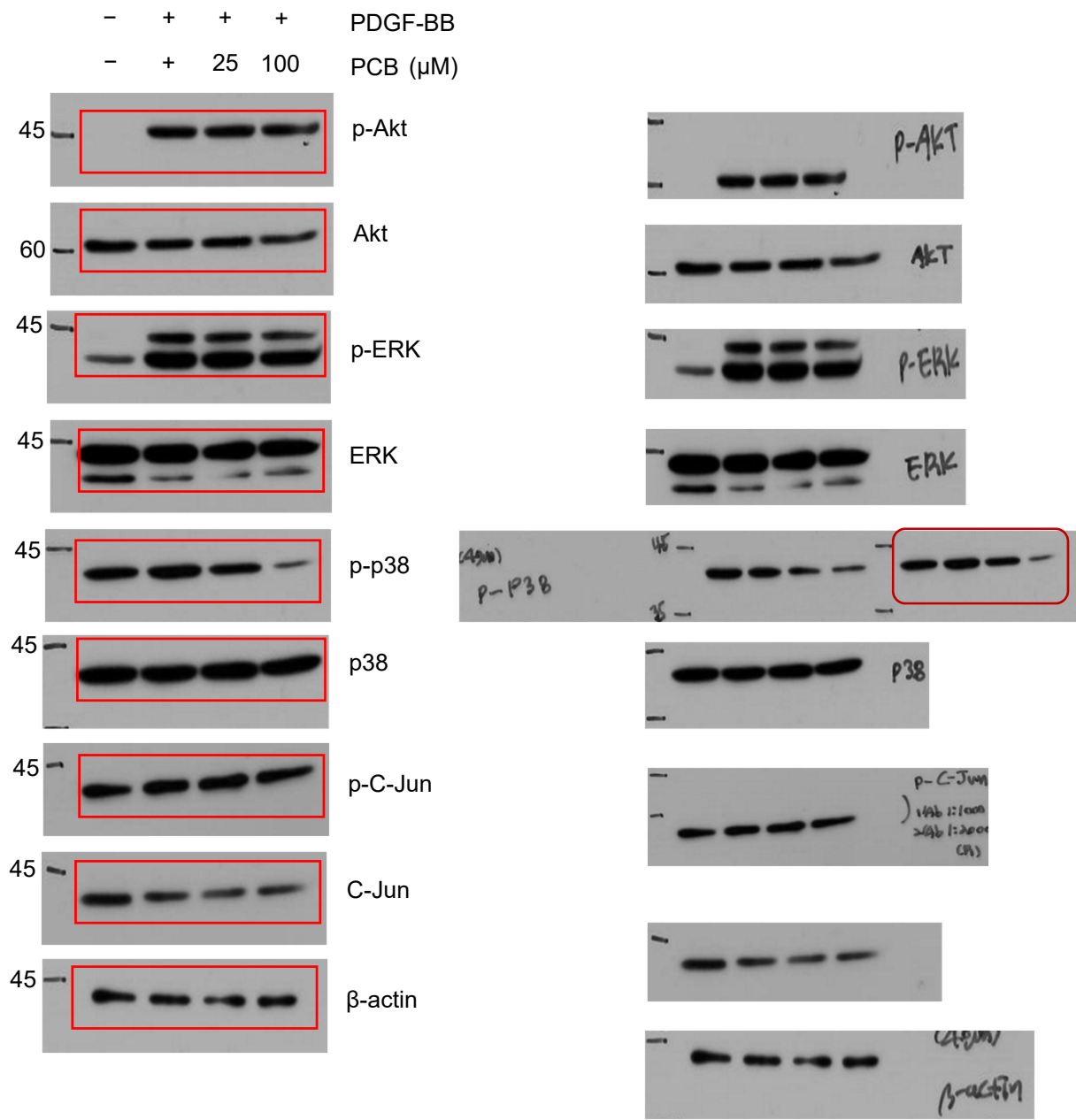

Supplement: Supplementary file 1 [file biomolecules-15-01325-s001.zip › biomolecules-3786371-supplementary.pdf]
